# The Influence of Retinol Ointment on Rabbit Skin (*Oryctolagus cuniculus*) Ion Transport—An In Vitro Study

**DOI:** 10.3390/ijms25179670

**Published:** 2024-09-06

**Authors:** Klaudia Dłubała, Sandra Wasiek, Patrycja Pilarska, Karolina Szewczyk-Golec, Celestyna Mila-Kierzenkowska, Krzysztof Z. Łączkowski, Marta Sobiesiak, Marcin Gackowski, Bartosz Tylkowski, Iga Hołyńska-Iwan

**Affiliations:** 1 Department of Pathobiochemistry and Clinical Chemistry, Faculty of Pharmacy, Ludwik Rydygier Collegium Medicum in Bydgoszcz, Nicolaus Copernicus University in Torun, 85-094 Bydgoszcz, Poland; 300773@cm.umk.pl (K.D.); 300770@cm.umk.pl (S.W.); 300815@cm.umk.pl (P.P.); 2 Department of Medical Biology and Biochemistry, Faculty of Medicine, Ludwik Rydygier Collegium Medicum in Bydgoszcz, Nicolaus Copernicus University in Torun, 85-092 Bydgoszcz, Poland; karosz@cm.umk.pl (K.S.-G.); celestyna_mila@cm.umk.pl (C.M.-K.); 3 Department of Chemical Technology and Pharmaceuticals, Faculty of Pharmacy, Ludwik Rydygier Collegium Medicum in Bydgoszcz, Nicolaus Copernicus University in Torun, 85-089 Bydgoszcz, Poland; krzysztof.laczkowski@cm.umk.pl; 4 Department of Inorganic and Analytical Chemistry, Faculty of Pharmacy, Ludwik Rydygier Collegium Medicum in Bydgoszcz, Nicolaus Copernicus University in Torun, 85-089 Bydgoszcz, Poland; marta.sobiesiak@cm.umk.pl; 5 Department of Toxicology and Bromatology, Faculty of Pharmacy, Ludwik Rydygier Collegium Medicum in Bydgoszcz, Nicolaus Copernicus University in Torun, 85-089 Bydgoszcz, Poland; marcin.gackowski@cm.umk.pl; 6 Eurecat, Technology Centre of Catalonia, Chemical Technology Unit, Marcelli Domingo 2, 43007 Tarragona, Spain; bartosz.tylkowski@eurecat.org; 7 Department of Clinical Neuropsychology, Faculty of Health Science, Ludwik Rydygier Collegium Medicum in Bydgoszcz, Nicolaus Copernicus University in Torun, 85-094 Bydgoszcz, Poland

**Keywords:** chloride, keratinocytes, skin, sodium, vitamin A, water transport

## Abstract

Retinoids are known to improve the condition of the skin. Transepithelial transport of sodium and chloride ions is important for proper skin function. So far, the effect of applying vitamin A preparations to the skin on ion transport has not been evaluated. In the study, electrophysiological parameters, including transepithelial electric potential (PD) and transepithelial resistance (R), of rabbit skin specimens after 24 h exposure to retinol ointment (800 mass units/g) were measured in a modified Ussing chamber. The R of the fragments incubated with retinol was significantly different than that of the control skin samples incubated in iso-osmotic Ringer solution. For the controls, the PD values were negative, whereas the retinol-treated specimens revealed positive PD values. Mechanical–chemical stimulation with the use of inhibitors of the transport of sodium (amiloride) or chloride (bumetanide) ions revealed specific changes in the maximal and minimal PD values measured for the retinol-treated samples. Retinol was shown to slightly modify the transport pathways of sodium and chloride ions. In particular, an intensification of the chloride ion secretion from keratinocytes was observed. The proposed action may contribute to deep hydration and increase skin tightness, limiting the action of other substances on its surface.

## 1. Introduction

**Figure 1:**
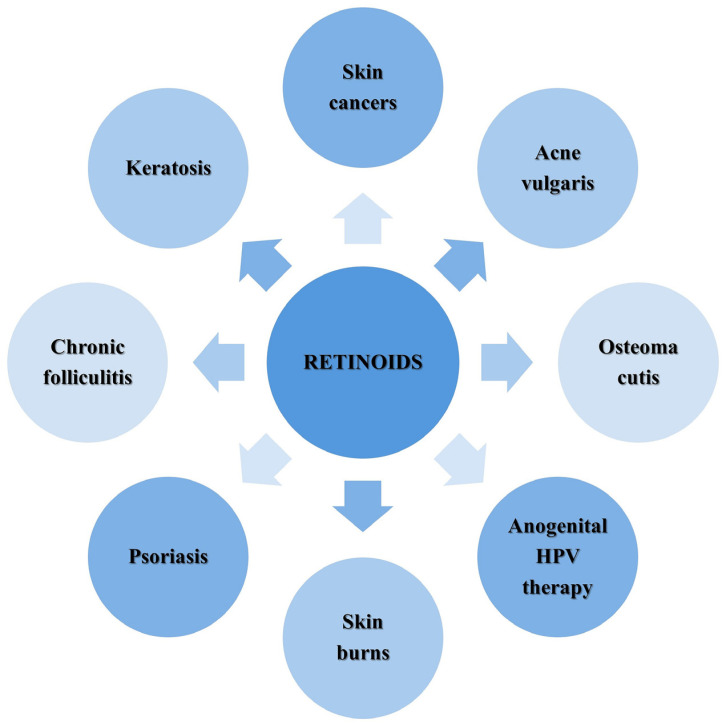
Skin conditions that may be treated with retinoids [1,3,4,5,7,10,19]. Abbreviations: HPV—human papilloma virus.

Vitamin A (Vit A) encompasses a group of molecules known as retinoids which includes retinol, retinal, retinoic acid, retinyl esters, and provitamin carotenoids [1,2,3,4,5]. They are essential micronutrients and play an extremely important role in many physiological processes in the body, including cell differentiation and proliferation, the perception of visual stimuli, bone growth, and immune responses [1,5,6,7,8,9,10]. Retinoids can bind to and activate retinoic acid receptors, which evolves specific biological responses [10,11,12,13,14]. Retinoid acid receptors (RARs) and retinoid X receptors (RXRs) are located in the nucleus of keratinocytes, fibroblasts, melanocytes, immunocompetent cells, follicle cells, and sebaceous glands [4,9]. In the dermis, retinoids activate fibroblasts [9,13,14,15,16], increase and/or regulate the production of collagen [2,8,11,15] and elastin [8,16,17,18], and stimulate endothelial cells, contributing to the formation of new vessels [5,16]. Moreover, they protect the formed collagen against degradation, increasing the elasticity and firmness of the skin and making it more durable and less susceptible to damage, as well as reducing wrinkles and eliminating skin discoloration [16,19,20], as shown in Figure 1. Furthermore, topical retinoids such as lipophilic compounds can penetrate into the stratum corneum, where they stimulate the proliferation of fibroblasts and keratinocytes [4,8,16,21]. This process leads to thickening of the outer layer of the skin and the exfoliation of dead cells [2], which contributes to strengthening the epidermal barrier and reducing transepithelial water loss (TEWL) [4,5,8,16,21]. Thus, not only have retinoids been wildly applied in cosmetics and wellness products [5,7,16,19], but they have also been considered in dermatology as active agents against skin diseases, such as acne, psoriasis, chronic inflammation of the hair follicles and sebaceous glands, melasma, ichthyosis, photodamage, and photoaging [2,5,13,16,20]. Topical retinoids that have gained widespread interest in cosmetology include tretinoin (all-trans retinoic acid), alitretinoin, retinol, retinol esters (retinyl acetate and retinyl palmitate), adapalene, bexarotene, and tazarotene (which belongs to the group of acetylated derivatives of retinoids) [11,16,17]. The concentration of retinol in cosmetic products ranges from 0.0015% to 0.3% [22]. In the case of topical tazarotene, its concentration ranges from 0.05% to 0.1%, while tretinoin used in anti-acne therapies is most often in the form of gels, creams, or liquid at a concentration of 0.1–0.4% [5].

Despite the positive effects of retinoids on the skin (Table 1), their use remains limited due to their side-effects. It has been shown that formulations containing retinoids may result in redness and irritation of the skin, the appearance of skin blemishes, or excessive peeling and drying [2,4,8,19,22].

One of the factors enabling the skin to perform many important functions is its proper hydration and the related transport of ions, mainly sodium and chloride [23,24,25,26]. Epithelial sodium channels (ENaCs) are responsible for the transport of sodium ions in the skin and, more specifically, in keratinocytes [25,26]. The transport of these ions is accompanied by the transport of water associated with the mechanism of equalization of osmolality between the intra- and extracellular space. Sodium ions flow into the cell upon the opening of an ENaC, taking water with them. Sodium transport stops when the channel is closed, and water moves to a space with a higher sodium concentration until the osmolality is equalized. The action of ENaCs modulates the immune response [23,24]. The cystic fibrosis transmembrane conductance regulator (CFTR) and other chloride channels are responsible for the transport of chloride ions within keratinocytes [27]. The presence of CFTR in the skin has been demonstrated in keratinocytes and sweat duct cells, where it participates in the regulation of the composition and amount of secreted sweat [27]. Substances that can change the activity of these channels are associated with the dehydration or hyperhydration of cells in individual layers of the skin and the extracellular environment [26]. CFTR also has a regulatory function and modulates the action of ENaCs [25,28]. Medicaments and xenobiotics applied and absorbed through the skin can affect the operation of sodium and chloride channels via their closing and opening, thus affecting the hydration of keratinocytes and the space surrounding them [29,30,31]. The effect of such action may include the occurrence of hypersensitivity reactions caused by the activation of immunocompetent cells and impaired wound healing and skin regeneration; moreover, pigmentation disorders may also occur [27,28,29,30,32]. The effect of vitamin A on the transport of chloride ions via the Na^+^/K^+^/Cl^−^ symporters (NKCCs) [33] and TEWL [22] has been proven.

So far, the effect of vitamin A and its derivatives on skin ion transport has not been evaluated. Therefore, the aim of this study was to assess the effect of retinol ointments on transepithelial ion transport, measured as transepithelial electric potential (PD) and transepithelial electric resistance (R). Based on the PD changes measured in stationary conditions and during stimulation (PDmin and PDmax) after the administration of retinol ointment, conclusions regarding the activity of sodium and chloride channels and changes in the hydration of the layers of the tested skin specimens have been formulated.

## 2. Results

The R initially measured at the beginning of the experiment for the fragments incubated with retinol was significantly lower than that measured for the controls (Mann–Whitney test, *p* < 0.001). The R measured at the end of the experiment was significantly lower (Wilcoxon test, *p* = 0.03) than that measured at the beginning within each Vit A group. For the control tissue samples not treated with retinol, no decrease in R was noted.

Similarly, the final R observed in the study group was significantly higher than that observed in the control group (Mann–Whitney test, *p* = 0.015). When comparing between the R measurements taken at the beginning of the experiment and those taken at the end of the experiment, we find that the R increased significantly after the administration of vitamin A under iso-osmotic conditions and incubation in Ami solution (Table 2, Mann–Whitney test). In the Wilcoxon test, we found a significant decrease in the R of skin fragments with vitamin A ointment incubated in Ami solution over the course of the experiment (Wilcoxon test, *p* < 0.001), which was not observed in the control specimens. In the case of the initial R measurement for tissues treated with vitamin A ointment and incubated in Bume solution, no statistically significant difference was observed compared to the control (Mann–Whitney test, *p* = 0.296). Over the course of this experiment, the R decreased significantly in the vitamin A group (Wilcoxon test, *p* = 0.002).

The PD value measured under stationary conditions for all control fragments did not change significantly during the experiment and remained constant regardless of the incubation conditions, which was confirmed by the Wilcoxon test (Table 3). Statistically significant changes in PD measured under stationary conditions over the course of the experiment were demonstrated in tissues with retinol incubated in amiloride and bumetanide, as proven by the Wilcoxon test (Table 3), in contrast to tissues with retinol incubated in RS, where no such changes were observed (Wilcoxon test, *p* = 0.798912). In the group of control tissues incubated in RS, the PD values were negative in contrast to the tissues with retinol, where the PD values were positive. The Mann–Whitney test showed a significant difference in the PD value between the control group and the study group (Mann–Whitney test, *p* < 0.001). In the groups of tissues incubated in solutions of amiloride and bumetanide, after the application of retinol, the appearance of an electronegative potential of the PD value was observed, in contrast to the control. In the case of incubation in bumetanide, a significant change was shown between the control and the study group, which was not demonstrated in the case of tissues incubated in Ami solution (Mann–Whitney test, Table 3).

Both the highest PDmax values (median: 0.87 mV) and the lowest PDmin values (median: −1.2 mV) were observed in tissues treated with retinol and incubated in RS. When comparing the PDmax values between the control tissues and retinol-treated tissues, for all incubations, statistically significant changes in this parameter were found (Mann–Whitney test, Table 4). The same was observed in the case of PDmin.

Using the Wilcoxon test, it was proven that the PD measured under stationary conditions (without stimulation) was significantly different from the potential measured during mechanical and mechanical–chemical stimulations. These changes apply to both the controls and the retinol-treated skin specimens, regardless of the incubation solution used (i.e., RS, Ami, or Bume). Each time a stimulus was applied, the stimulation caused reproducible and measurable changes in the potential for all tested skin fragments (Wilcoxon test, Table 5).

## 3. Discussion

Vitamin A and its derivatives affect the immune system, scavenge free radicals, support the vision process, regulate erythropoiesis in the bone marrow, and also affect the proper development of reproductive cells and embryos, as well as skin functions [1,7,20]. In recent years, the use of retinoids on the skin has increased, especially in the treatment of acne and psoriasis, but also in skin care and anti-aging cosmetics [4,5,16,19]. The skin is a very important organ that protects against the harmful effects of external factors, taking part in the immune response and thermoregulation; it is also an organ of the senses [16]. One of the processes enabling the maintenance of proper skin functions is the constantly occurring transport of ions in skin cells, involving the secretion of chlorides and absorption of sodium, and the related transport of water inside and outside keratinocytes [25,26]. The transport of ions and water is possible due to the functioning of ENaCs, CFTR, NKCCs, aquaporins, and other channels and transporters [23,24,25,26,27,31]. Ion transport in the skin can be reflected in the measurement of skin PD and R [25,26,30]. The modified Ussing apparatus is a tool for assessing the transport of ions and water in epithelial tissue samples after their exposure to chemicals [25,26,30,31]. Modification of the Ussing chamber, in which skin fragments can be placed in a horizontal position, enables the application of a mechanical stimulus to the top layer of the skin [25,26,30,31]. The measurement of PD and R makes it possible to assess the pathomechanisms of diseases occurring with changes in the hydration of skin tissue and impairment to the function of, among other things, ion channels [25]. In this study, fragments of rabbit skin with a preserved layered structure and appropriate thickness with present nerve endings [33] were subjected to experimentation. Such structure of the skin enabled the estimation of ion transport in the skin treated with retinol ointment after stimulation with solutions of sodium (amiloride) and chloride (bumetanide) ion inhibitors [25,26,30,31].

The R of the skin reflects its physiological condition, its cell vitality, and its ability to pass ions [25,26,33]. The resistance depends, among other things, on the hydration of the cells and the continuity of the skin, the thickness of the epidermis, and the activity of immunocompetent cells [25,26,30]. Measurement of the resistance both at the beginning and at the end of the experiment showed that all analyzed fragments were viable and showed no damage in all experimental groups (Table 2, Wilcoxon test).

During the incubation of tissues treated with retinol ointment in both iso-osmotic RS and Ami solutions, the value of R significantly increased compared to the control fragments (Table 2). Such a high R value was most likely due to the sealing of the spaces between the cells by the retinol ointment, their good adhesion, and the limited ion transport. Thus, it can be assumed that the retinol ointment acted as another sealing layer. In the case of the inhibited transport of Cl^−^ ions (i.e., in the incubation with Bume solution), no significant increase in resistance between the tissues of the control and study groups was demonstrated (Table 2, Mann–Whitney test), which may indicate that retinol might limit the transport of sodium ions.

For the skin specimens treated with retinol, the R measured after 20 min of the experiment decreased significantly compared to the initial R measurement. There was no decrease in R for the control fragments (Table 2, Wilcoxon test). The applied stimulation flushed the ointment from the surface of the tissue and/or caused more intense penetration into the cells. However, during the incubation in Bume solution, with the inhibition of the Cl^−^ ion transport pathway, this effect was not observed for tissues treated with retinol. Zhang et al. [34] proved that retinoids inhibit the NKCC transporter. In tissues treated with bumetanide, the NKCC transporter was already inhibited; therefore, the vitamin A used did not show any inhibitory effect. Studies by Babina et al. [6] and Shao et al. [3] have shown that vitamin A can penetrate into the cells two hours after administration in a manner dependent on the demand of keratinocytes for Vit A. Unabsorbed vitamin A remains available to other cells in the intercellular spaces until the skin layer is shed. In addition, vitamin A can affect the preservation of physiological intercellular spaces [3]. Sealing the epidermis could be one of the reasons for the decreased intensity of ion transport measured by the increase in skin resistance.

For control tissues, the PD did not change significantly during the experiments and remained constant regardless of the incubation conditions (Wilcoxon test, Table 3). For the skin fragments treated with Vit A ointment, electropositive values were noted, regardless of the incubation conditions used, which was not observed for the controls. This may indicate the penetration of vitamin A into the cells and its local effect on minimizing mainly the transport of sodium ions. For both the control and retinol-treated fragments, the PD comparison showed significant differences, regardless of the incubation conditions used. On the other hand, incubation in ion transport inhibitors (Ami and Bume solutions) induced a similar direction of changes in PD for the controls and vitamin A group (Table 3, Mann–Whitney test). It can be assumed that retinol does not change the constantly occurring transport of Na^+^ and Cl^−^ ions, i.e., the function of the sodium–potassium pump and channels that maintain the constantly occurring transport of sodium and potassium ions. The PD of skin fragments lubricated with retinol ointment, measured at the beginning and end of the experiment, was significantly higher in the conditions without the use of ion transport inhibitors (Table 3, Mann–Whitney test, PD initial/final control vs. PD initial/final retinol). This may indicate the penetration of vitamin A into the cells [3] and its local effect on the transport of ions. After incubation in bumetanide, the PD significantly decreased compared to the control, intracellular sodium transport was minimized, and the secretion of chloride ions increased.

Reducing the intracellular transport of sodium ions and their accumulation with water in the intercellular spaces may be associated with the effect of vitamin A on the release of immunomodulatory proteins from keratinocytes [35] and the reduction in TEWL [5,20,21]. The even distribution of proteins released by keratinocytes between skin cells may contribute to the regulation of the influx of immunocompetent cells and improve their functioning. In addition, the creation of hydrated micro-spaces promotes the transport and assembly of pro-collagen chains, which is the cause of the long-term effect of sealing and firming of the skin due to the action of vitamin A [2]. It seems that the proposed 24 h skin contact with vitamin A is sufficient to initiate these reactions.

For tissues incubated in solution Ami, this phenomenon was not observed; therefore, the effect on the constantly occurring Cl^−^ transport was weaker. Retinol did not cause increased chloride secretion through the CFTR channel and other chloride channels present in keratinocytes, probably due to the inhibition of the NKCC transporter by vitamin A and thus the prevention of the intracellular transport of chlorides [34]. The retention of water in the spaces around the cells helps to retain substances that are present in the skin.

The applied stimulation caused changes in potential for all tested skin fragments. The results of the Wilcoxon test show that the PD values were significantly different from the potential values measured in conditions of mechanical and mechanical–chemical stimulation, i.e., the PDmin and PDmax values (Table 5). The use of retinol did not inhibit the transport of ions under the influence of mechanical stimuli in the analyzed system. Retinol ointment did not reduce the ability to perceive stimuli through the skin in conditions without the use of ion transport blockers. For both the incubation in RS and the incubation in Ami solution, the reactions to mechanical/mechanical–chemical stimuli measured by the changes in PDmax and PDmin were more intense after the administration of retinol. In the phase of increased secretion of chloride ions (Ami solution), the reactions were more intense. On the other hand, the inhibition of the chloride ion transport pathway with Bume solution caused a reduced reaction to the mechanical stimuli, i.e., close to that of the control specimens. For the control tissues, a comparison of the reaction to the mechanical stimulus with the reaction to the Vit A stimulation revealed significant differences in the reduction in PDmax and PDmin, while for tissues treated with retinol, this effect was not observed. It can be concluded that retinol acted primarily on the increase in Cl^−^ secretion and minimization of Na^+^ transport, which occurred in response to stimuli. The increase in chloride secretion and minimal inhibition of sodium adsorption under the influence of mechanical stimuli cause the accumulation of water in the microspaces around the cells [27]. Maintaining hydration improves the transport of nutrients [3], immunomodulatory substances [23], or metabolites and reduces the effect of xenobiotics, i.e., drugs, that come into contact with the skin.

The main limitation of our experiment is the use of rabbit skin. Despite various similarities, it has different properties than human skin, such as a greater number of hair follicles [33]. Unfortunately, human skin at full thickness, healthiness, and reactivity is difficult to access. Moreover, taking human skin for basic research is ethically controversial. However, the structure and properties of rabbit and human skin are similar enough that the observed results, e.g., the changes in transepithelial electric potential and resistance, which are the result of ion and water transport through the tested tissue specimens, can be used in clinical practice.

In our opinion, the greatest value of the experiments conducted in this study lies in the use of living skin, unaffected by any disease, fully reactive, and with preserved nerve endings and functioning cells that build the skin. Undoubtedly, the changes in ion and water transport under the influence of retinol ointment found in the performed in vitro study can be translated into the processes occurring in vivo.

## 4. Materials and Methods

This study of electrophysiological parameters was performed on rabbit skin from the inner part of the ear. The experiment was carried out on 90 pieces of skin taken from eight New Zealand White rabbits. Scarification of the animals was performed with 60% carbon dioxide. The death of an animal was confirmed through two methods by a qualified person.

The following reagents and solutions were used in this experiment:

- RS—Ringer’s solution: K^+^ 4.0 mM; Na^+^ 147.2 mM; Ca^2+^ 2.2 mM; Mg^2+^ 2.6 mM; Cl^−^ 160.8 mM; 4-(2-Hydroxyethyl)piperazine-1-ethanesulfonic acid (Sigma-Aldrich, USA). Iso-osmotic basic solution. Used to incubation and mechanical stimulation.
- Ami—amiloride 0.1 mM (3,5-diamino-6-chloro-2-carboxylic acid) 266.09 g/mol (Sigma-Aldrich, USA). Used as an inhibitor of the sodium ion transport pathway in incubation and mechanical–chemical stimulation tests.
- Bume—bumetanide 0.1 mM (3-butylamino-4-phenoxy-5-sulfamoylbenzoic acid) 364.42 g/mol (Sigma-Aldrich, USA). Used as an inhibitor of the chloride ion transport pathway in incubation and mechanical–chemical stimulation tests.
- Retinol—retinol palmitate. Ointment with retinol at a concentration of 800 mass units/g (Hasco-Lek S.A., Wrocław, Poland). Used in incubation tests.

### Experimental Procedure

**Figure 2:**
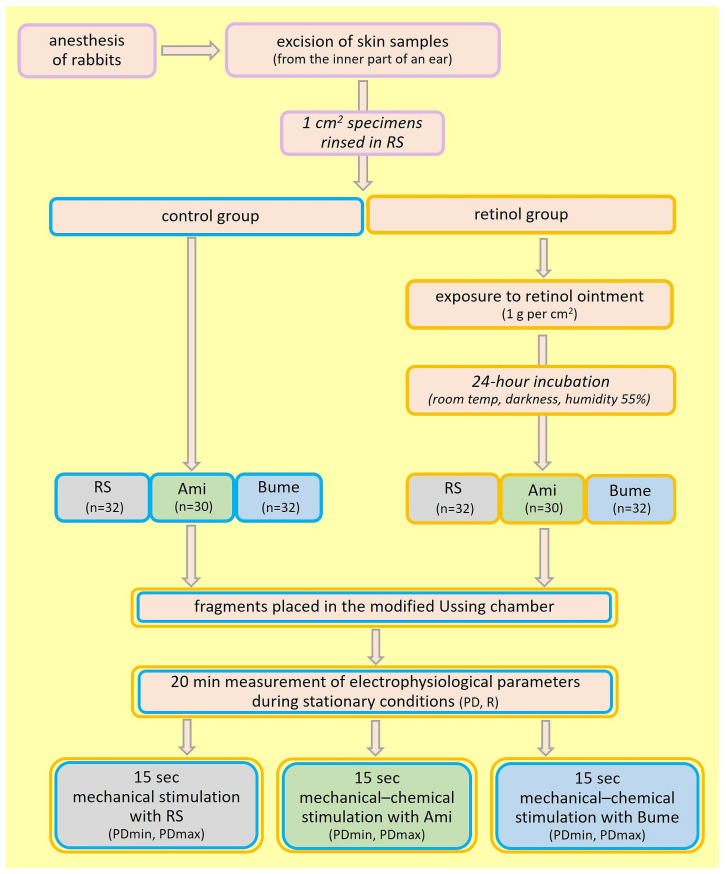
Study design. Abbreviations: RS—iso-osmotic Ringer solution; Bume—bumetanide (0.1 mM) solution; Ami—amiloride (0.1 mM) solution; PD—transepithelial electric potential measured in stationary conditions (mV); PDmax—maximal transepithelial electric potential measured during 15 s of mechanical or mechanical–chemical stimulation (mV); PDmin—minimal transepithelial electric potential measured during 15 s of mechanical or mechanical–chemical stimulation (mV); R—transepithelial resistance (Ω/cm^2^).

The examined skin specimens were cleaned and rinsed in RS, then exposed to the action of retinol ointment (1 g per 1 cm^2^) and left for 24 h at room temperature in the darkness, with a constant humidity of 55%. After this time, the analyzed skin fragments were placed in a horizontal position in a modified Ussing chamber filled with an incubation solution. The tested skin surface was 1 cm^2^. The modification of the chamber consisted of performing a series of stimulations of the stratum corneum with a liquid from a peristaltic pump with a fixed flow of 0.06 mL/s (1 mL/15 s). This research model imitated freely falling drops on the surface of the examined tissue. After 2 min, the electrophysiological parameters of the skin stabilized, and a series of stimulations was performed according to the experimental protocol (Figure 2). The experiment lasted 20 min for each fragment.

These experiments consisted of measuring the following parameters:

- PD—transepithelial electric potential measured continuously under stationary conditions (mV);
- PDmin and PDmax—minimal and maximal transepithelial electric potential measured during 15 s of mechanical and/or mechanical–chemical stimulation (mV);
- R—transepithelial resistance measured after applying a stimulus current of ±10 μA to the tissue (after measuring the voltage, the resistance was calculated according to Ohm’s law (Ω/cm^2^)).

Data were recorded using the EVC4000 experimental protocol (WPI, Worcester, MA, USA), which was connected to the data acquisition system and transferred to the AcqKnowledge 3.8.1 computer software (Biopac Systems, Inc., Goleta, CA, USA). Statistical analyses were performed in Statistica 11.00 (StatSoft, Polska, Kraków, Polska). In order to determine the data distribution, the Kolmogorov–Smirnov test, with Lilefors corrections, was used. The Wilcoxon test and the Mann–Whitney test were also used, with *p*-values < 0.05 denoting significance.

## 5. Conclusions

Importantly, retinol palmitate seemed to seal the spaces between keratinocytes, causing an increase in skin resistance. In addition, we observed an intensification of the chloride ion secretion from keratinocytes after the mechanical stimulus was applied. However, the observed ion transport was limited to small spaces around the cells, favoring the accumulation of water. The proposed action may contribute to deep moisturizing and increased tightness of the skin, limiting the action of other substances present on its surface. This can help to smooth the skin surface and normalize the keratinization, which is especially important for patients whose skin barrier is disturbed due to skin diseases.

## Figures and Tables

**Table 1:** Effects of vitamin A on human skin.

		EFFECTS OF VITAMIN A ON HUMAN SKIN	
		Major Cells	Action
**SKIN LAYER**	**EPIDERMIS**	Keratinocytes	Exfoliation of dead cells Proliferation of live cells Strengthening of the epidermal barrier Mitigation of TEWL
	**DERMIS**	Fibroblasts	Activation of fibroblast production Stimulation of fibroblasts Elevation of the production of collagen and elastin Protection of collagen destruction by affecting the synthesis of tissue inhibitors of metalloproteinases
		Endothelial cells	Synthesis of new capillary networks

Abbreviations: TEWL—transepidermal water loss.

**Table 2:** Transepithelial electric resistance (R) measured under stationary conditions for skin specimens treated with retinol ointment in 3 study groups (RS, Ami, Bume) and control skin specimens in 3 control groups (RS, Ami, Bume).

		Control		Wilcoxon Test (*p*) Control	Retinol		Wilcoxon Test (*p*) Retinol	Mann–Whitney Test (*p*)	
**Incubation**		**R Initial (** Ω **/cm^2^)**	**R Final (** Ω **/cm^2^)**	**R Initial vs. R Final**	**R Initial (** Ω **/cm^2^)**	**R Final (** Ω **/cm^2^)**	**R Initial vs. R Final**	**R Initial:** **Control vs. Retinol**	**R Final:** **Control vs. Retinol**
**RS** (n = 32)	Median	11,779	12,907	0.059	34,770	30,765	0.030	<0.001	0.015
	Lower quartile	5417	6565		13,786	4580			
	Upper quartile	28,492	28,891		68,681	61,651			
**Ami** (n = 30)	Median	3101	3108	0.594	27,525	23,368	<0.001	0.004	0.005
	Lower quartile	2202	1959		4473	3784			
	Upper quartile	6700	3989		67,306	62,322			
**Bume** (n = 32)	Median	11,759	10,904	0.629	20,941	19,773	0.002	0.296	0.390
	Lower quartile	4857	4141		4991	5130			
	Upper quartile	31,662	31,728		43,416	35,878			
**Mann–Whitney test (*p*)**	RS vs. Ami	0.011	0.003		0.465	0.500			
	RS vs. Bume	0.994	0.733		0.021	0.044698			
	Ami vs. Bume	0.009	0.003		0.287	0.378340			

Abbreviations: R—resistance (Ω/cm^2^); RS—iso-osmotic Ringer solution; Bume—bumetanide 0.1 mM solution; Ami—amiloride 0.1 mM solution; control—skin specimens incubated with RS, Ami, or Bume solution; retinol—skin specimens treated with ointment containing retinol palmitate 1 g/cm^2^ for 24 h and incubated with RS, Ami, or Bume solution; n—number of specimens. Significance level: *p* < 0.05.

**Table 3:** Transepithelial electric potential (PD) measured under stationary conditions for skin specimens treated with retinol ointment in 3 study groups (RS, Ami, Bume) and control skin specimens in 3 control groups (RS, Ami, Bume).

		**Control**		**Wilcoxon Test (*p*) Control**	**Retinol**		**Wilcoxon Test (*p*)** **Retinol**	**Mann–Whitney Test (*p*)**	
**Incubation**		**PD Initial (mV)**	**PD Final (mV)**	**PD Initial vs. PD Final**	**PD Initial (mV)**	**PD Final (mV)**	**PD Initial vs. PD Final**	**PD Initial:** **Control vs. Retinol**	**PD Final:** **Control vs. Retinol**
**RS** **(n = 32)**	Median	−0.22	−0.25	0.220473	0.11	0.18	0.798912	<0.001	<0.001
	Lower quartile	−0.56	−0.40		−0.07	−0.09			
	Upper quartile	0	0		0.56	0.49			
**Ami** **(n = 30)**	Median	0	0	0.399309	−0.14	−0.04	0.037199	0.080973	0.853131
	Lower quartile	−0.21	−0.24		−0.34	−0.18			
	Upper quartile	0.18	0.13		0.05	0.07			
**Bume** **(n = 32)**	Median	0.32	0.37	0.127114	−0.24	−0.13	<0.001	0.002119	0.001038
	Lower quartile	−0.15	0		−0.47	−0.37			
	Upper quartile	0.43	0.49		−0.03	−0.04			
**Mann–Whitney test (*p*)**	RH vs. Ami	0.002452	0.078170		< 0.001	0.006976			
	RH vs. Bume	<0.001	<0.001		< 0.001	< 0.001			
	Ami vs. Bume	0.137087	0.135974		0.422000	0.220566			

Abbreviations: PD—transepithelial electric potential measured in stationary conditions (mV); RS—iso-osmotic Ringer solution; Bume—bumetanide 0.1 mM solution; Ami—amiloride 0.1 mM solution; control—skin specimens incubated with RS, Ami, or Bume solution; retinol—skin specimens treated with ointment containing retinol palmitate 1 g/cm^2^ for 24 h and incubated with RS, Ami, or Bume solution; n—number of specimens. Significance level: *p* < 0.05.

**Table 4:** Maximal (PDmax) and minimal (PDmin) transepithelial electric potential measured during 15 s of mechanical or mechanical–chemical stimulation for skin specimens in the control and retinol groups.

		Control		Retinol		Mann–Whitney Test (*p*)	
Stimulation		PDmax (mV)	PDmin (mV)	PDmax (mV)	PDmin (mV)	PDmax: Control vs. Retinol	PDmin: Control vs. Retinol
**RS (n = 32)**	Median	0.87	−0.5	1.60	−1.2	0.038378	0.074359
	Lower quartile	0.21	−1.07	0.55	−2.64		
	Upper quartile	2.72	−0.21	4.33	−0.03		
**Ami (n = 30)**	Median	0.21	−0.29	1.575	−0.77	<0.001	<0.001
	Lower quartile	0.00	−1.1	0.34	−3.11		
	Upper quartile	1.04	0.00	4.85	−0.43		
**Bume (n = 32)**	Median	1.95	−0.55	0.58	−0.82	0.003968	0.024506
	Lower quartile	1.07	−1.83	0.46	−1.63		
	Upper quartile	6.41	0.15	5.66	−0.37		
**Mann–Whitney test (*p*)**	RH vs. Ami	0.003475	0.038445	0.646064	0.445383		
	RH vs. Bume	0.004527	0.637409	0.102272	0.335190		
	Ami vs. Bume	<0.001	0.140427	0.065451	0.101114		

Abbreviations: PDmax—maximal transepithelial electric potential measured during 15 s of mechanical or mechanical–chemical stimulation (mV); PDmin—minimal transepithelial electric potential measured during 15 s of mechanical or mechanical–chemical stimulation (mV); RS—iso-osmotic Ringer solution; Bume—bumetanide 0.1 mM solution; Ami—amiloride 0.1 mM solution; control—skin specimens incubated with RS, Ami, or Bume solution; retinol—skin specimens treated with ointment containing retinol palmitate 1 g/cm^2^ for 24 h and incubated with RS, Ami, or Bume solution; n—number of specimens. Significance level: *p* < 0.05.

**Table 5:** Results of the Wilcoxon test for skin specimens in the control and retinol ointment groups regarding transepithelial electric potential measured in stationary conditions (PD) and maximal (PDmax) and minimal (PDmin) transepithelial electric potential measured during 15 s of mechanical or mechanical–chemical stimulation.

	Control			Retinol		
Parameters	RS	Ami	Bume	RS	Ami	Bume
**PD vs. PDmax**	<0.001	<0.001	<0.001	<0.001	<0.001	<0.001
**PD vs. PDmin**	<0.001	<0.001	<0.001	<0.001	<0.001	<0.001
**PDmax vs. PDmin**	<0.001	<0.001	<0.001	<0.001	<0.001	<0.001

Abbreviations: PD—transepithelial electric potential measured in stationary conditions (mV); PDmax—maximal transepithelial electric potential measured during 15 s of mechanical or mechanical–chemical stimulation (mV); PDmin—minimal transepithelial electric potential measured during 15 s of mechanical or mechanical–chemical stimulation (mV); RS—iso-osmotic Ringer solution; Bume—bumetanide 0.1mM solution; Ami—amiloride 0.1mM solution; control—skin specimens incubated with RS, Ami, or Bume solution; retinol—skin specimens treated with ointment containing retinol palmitate 1 g/cm^2^ for 24 h and incubated with RS, Ami, or Bume solution. Significance level: *p* < 0.05.

## Data Availability

This study’s data will be made available if requested; for such inquiries, please email igaholynska@cm.umk.pl.
